# Diabetes: Its Cause and Permanent Cure

**Published:** 1889-09

**Authors:** 


*Diabetes: its Cause and Permanent Cure.* By EMIL SCHNÉE, M.D. Translated from the German by R. L. TAFEL, A.M., Ph.D. London: H. K. LEWIS. 1889.

This book is of a quality which, to the credit of Medicine be it said, is happily seldom met with. To make it the subject of a serious review is undesirable even if it were possible, for the work must be read to be believed; but as it should not be believed, neither therefore should it be read.

The author may be allowed one quotation by way of displaying his views and style (the italics are his, not ours):

"*Herein lies the great mystery, never before discovered by anyone, of the cause of 'diabetes,' and here, according to my conviction, hereditary lues on the part of one of the parents, grand-parents, or great-grand-parents of the patient has to be taken into consideration. A transmission of lues also through vaccination is not excluded.*"

Perhaps after all the book is a portentous joke, the point of which has not been perceived by the solemn Teutonic translator. The literary history and bibliography of Diabetes is fairly well done.

